# Autoimmune Hepatitis: A Review of Molecular Mechanisms and Research Gaps in African Populations

**DOI:** 10.3390/biology15050400

**Published:** 2026-02-28

**Authors:** Caitlin Wheeler, Janine Scholefield, Tracey Hurrell, Jerolen Naidoo

**Affiliations:** 1 Bioengineering and Integrated Genomics Group, Future Production Chemicals Cluster, Council for Scientific and Industrial Research, Pretoria 0184, South Africa; 2 Division of Human Genetics, National Health Laboratory Service and School of Pathology, Faculty of Health Sciences, University of the Witwatersrand, Johannesburg 2001, South Africa; 3 Wits Donald Gordon Medical Research Institute, Faculty of Health Sciences, University of the Witwatersrand, Johannesburg 2193, South Africa; 4 Department of Pharmacology, Faculty of Health Sciences, University of Pretoria, Pretoria 0184, South Africa; 5 Department of Biochemistry, Genetics and Microbiology, Faculty of Natural and Agricultural Sciences, University of Pretoria, Pretoria 0184, South Africa; 6 Department of Human Biology, Faculty of Health Sciences, University of Cape Town, Cape Town 7700, South Africa

**Keywords:** autoimmune hepatitis, autoimmunity, molecular mechanisms, pathogenesis, Africa

## Abstract

Autoimmune hepatitis (AIH) is an inflammatory liver disease characterised by immune-mediated hepatic injury, often leading to liver failure. The underlying molecular mechanisms of AIH remain poorly elucidated, hindering diagnostic and therapeutic advances. This review overviews the current understanding of AIH pathogenesis, which arises from a complex interplay of genetic predisposition, environmental triggers, and immune mechanisms (loss of tolerance, regulatory T cell dysfunction). Furthermore, current technologies and models which are being used to deconvolve the molecular profiles and pathophysiology of AIH are also discussed. Although AIH has a low reported global burden, AIH research is critically skewed towards European ancestry populations. This leaves a significant knowledge gap in diverse ancestry groups, such as those of African ancestry, where emerging research suggests that these patients may experience a more aggressive disease. Collectively, this highlights the need for research in underrepresented global populations to develop tailored diagnostics and effective targeted treatments.

## Simple Summary

Autoimmune hepatitis is a liver disease where the body’s immune system attacks its own liver cells because it no longer recognises them, causing inflammation and damage that can eventually lead to liver failure. We do not fully understand the exact steps that cause autoimmune hepatitis, which makes it hard to figure out if a person has it and how best to treat it. This paper reviews what we currently know about how autoimmune hepatitis starts. It is a complicated mix of an individual’s genes, things they encounter in their environment, and the immune system. The review also looks at new technologies and models which scientists are using to figure out the precise biological details of the disease. Crucially, research on autoimmune hepatitis has focused almost entirely on people of European descent. This leaves a huge gap in our knowledge for other global groups, especially those of African ancestry, where evidence suggests they might experience a more severe form of the disease. Therefore, future research must focus on these underrepresented populations to develop tests and treatments that work well for everyone.

## 1. Introduction

Autoimmune hepatitis type 1 (AIH-1 or AIH) is a chronic inflammatory liver disease characterised by immunopathogenic targeting and destruction of hepatocytes. If left untreated, this autoimmune disease can lead to irreversible hepatic injury and liver failure [1,2,3]. The diagnosis and treatment of AIH are complicated by phenotypic heterogeneity in clinical presentation and poorly elucidated mechanisms underlying disease aetiology and progression. The clinical hallmarks include elevated liver enzymes, the presence of serum autoantibodies, and histological evidence of interface hepatitis [1,2,3,4,5].

Research on liver diseases, including AIH, has predominantly occurred in developed regions, resulting in a representation bias in which populations of European ancestry predominate [4,6]. Emerging research in North America (particularly Hispanic and African American ancestry populations), Asia, and South America investigate the prevalence, genetic predispositions, and clinical presentations of AIH in these diverse and historically under-researched populations [1,7,8,9,10,11]. However, there remains a significant knowledge gap in AIH research in Africa, where autoimmune and metabolic diseases are commonly misdiagnosed [12].

The current lack of understanding of the molecular mechanisms of AIH has hindered the potential for diagnostic advances and development of targeted treatment strategies [4]. This review aims to provide an overview of the current understanding of the molecular basis of AIH while highlighting the need to conduct research in underrepresented populations, such as in Africa.

## 2. Epidemiology

### 2.1. Incidence and Prevalence

As a rare autoimmune liver disease, AIH has a global pooled incidence of less than 1.5 per 100,000 people per year with varied reports across geographic population groups [6,13]. In North America and Denmark, AIH is more frequently diagnosed, with incidence rates of ~4–5 per 100,000 people per year [6,14,15,16]. In Asia, the pooled incidence of AIH is ~1 case per 100,000 suggesting a lower reported incidence; however, data from Asia remains sparse [6,17].

The global pooled prevalence of AIH is estimated to be 15–17 per 100,000 people, with notably higher prevalence recorded in regions with greater diagnostic capabilities, such as North America (estimated at 29 per 100,000 people) [6,13,18]. However, studies from Japan and China suggest an increasing scientific and clinical interest in AIH in these populations with a pooled prevalence of ~8–12 per 100,000 persons reported [6,9,13,19]. These geographical disparities may be reflective of underdiagnosis, differing diagnostic capabilities, and limited research rather than an actual lower disease burden.

### 2.2. Risk Factors

#### 2.2.1. Sex

The complex interplay of genetics (sex chromosomes), sex steroid hormones, epigenetics, and other factors is likely to influence the sex biases observed in the epidemiology and immune responses of autoimmune diseases [20,21,22,23]. A strong sex-based bias towards female individuals has been observed in autoimmune diseases such as systemic lupus erythematosus, Sjögren’s syndrome, systemic sclerosis, and AIH [21,22]. Epidemiological studies on AIH suggest that approximately 70–80% of AIH patients are female [1,6,14,16,20,22,24,25]. This female predominance aligns with findings linking dosage-sensitive X-linked genes and oestrogen fluctuations to sex-biased autoimmunity [26,27,28].

It has been hypothesised that having more than one X chromosome increases autoimmunity risk due to the gain of X-linked immunity genes, whereby some of these dosage-sensitive genes escape the process of X-inactivation [26,28,29]. An example of a dosage-sensitive X-inactivation-escaping gene implicated in autoimmune disease is Forkhead box P3 (*FOXP3*) [21]. This critical transcription factor is primarily involved in the development and function of regulatory T cells (Tregs), providing a mechanistic link between immune gene dosage and immune response phenotypes [21]. Furthermore, upregulation of oestrogen receptor alpha (*ERα*) has been shown to impair Treg function in pre-menopausal females diagnosed with AIH, demonstrating that hormone signalling pathways may also be implicated in the observed sex bias [30]. Additionally, there are sex-based differences in cell subtype composition and phenotype across the innate and adaptive immune systems [23]. Specifically of interest, Tregs have a lower number and less robust response in females in comparison to males [23]. However, within AIH, the potential impact of sex biases on T cell, B cell, neutrophil, and monocyte populations still needs further research.

Varying levels of hormones, such as oestrogen and testosterone, during different environmental and age-related stages have been shown to impact immunity [27]. Oestrogen, which is more abundant in females, enhances immune activation, intensifying responses from T and B cells [27]. Increased pregnancy-induced oestrogen levels, peaking in the third trimester, are hypothesised to play a role in disease onset and progression of AIH. During maternities, AIH and metabolic dysfunction-associated steatotic liver disease (MASLD) have been reported as the most common underlying aetiologies of liver cirrhosis [31,32]. Additionally, there are higher observed rates of gestational diabetes, hypertensive disorders, pre-term births, and foetal growth restrictions in pregnant AIH patients [5]. In contrast, androgens like testosterone, a sex hormone more abundant in males, generally suppress immune responses and, to date, have not been investigated in association with AIH. Furthermore, environmental and behavioural factors, such as smoking and antibiotic use, can interact with sex hormones to affect immune regulation and autoimmune risk in a sex-specific manner [33,34,35].

Sex biases are observed in the gene expression profiles of male and female healthy liver tissue donors, with further cell type-specific differences also being distinguishable [36]. For example, Hepatocyte Growth Factor (*HGF*), which stimulates cell growth/motility and is critical for organ development and regeneration, was enriched at a young age (18–40 years) and in males [36]. Angiopoietin-2 (*ANGPT2*), which regulates angiogenesis and inflammation, was upregulated at a young age (18–40 years) and in females [36]. Therefore, considering the 1:4 male–female ratio observed for AIH, understanding the contextualised and tissue-specific sex differences in immunity is critical in assessing the disease epidemiology, aetiology, diagnosis, and treatment.

#### 2.2.2. Age

Diagnosis occurs most often in young-to-middle-aged adults, but is not exclusive to this age range, as disease onset is also observed in children, adolescents, and elderly adults [4,15]. Menopause is suggested to influence the age distribution of AIH in women, with a notable increase in diagnoses in perimenopausal women, potentially due to hormonal changes that impact immune regulation [20,21,22]. Studies in paediatric and adult cohorts across different ethnicities (including from Sub-Saharan African and Brazilian populations) have shown a lower average age of diagnosis in comparison to populations of European ancestry [37,38,39,40,41]. In elderly patients (≥60 years), AIH is commonly misdiagnosed [1,42,43,44,45,46]. Nevertheless, an increasing incidence of AIH in older adults has been observed in European, Latin American and Asian populations, with few-to-no published reports in African populations [42,43,44,45]. The broad age range of AIH onset suggests that both genetic predispositions and environmental factors could contribute to disease aetiology.

## 3. Pathophysiology

**Figure 1:**
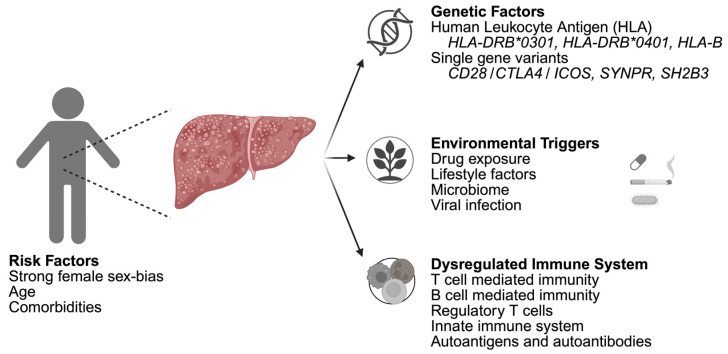
Pathophysiology of autoimmune hepatitis illustrating the influence of risk factors, genetic predisposition, environmental triggers, and the disruptive effect on the cellular components of the immune system. Figure created in Biorender.com.

Autoimmune hepatitis is known to be driven by a dysregulated immune response where hepatocytes are erroneously targeted [1,4]. The exact molecular mechanisms are not fully elucidated but are influenced by genetic predisposition, environmental factors, and the disruption of immunological homeostasis (Figure 1) [1,4]. The interplay of these factors results in chronic inflammation and progressive hepatic damage.

### 3.1. Genetic Factors

Genetic predisposition plays a critical role in the pathophysiology of AIH, with research highlighting the involvement of human leukocyte antigen (*HLA*) genes alongside other novel loci increasingly being implicated in disease susceptibility and progression.

#### 3.1.1. Human Leukocyte Antigen

**Figure 2:**
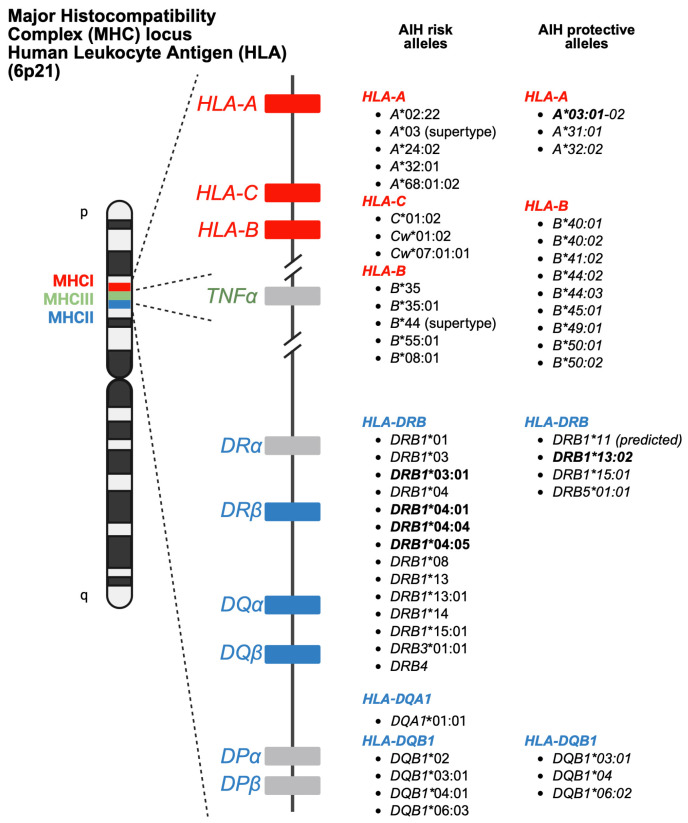
Human Leukocyte Antigen (*HLA*) alleles conferring risk and protecting against autoimmune hepatitis. Alleles highlighted in bold are discussed in detail in the main text. Figure created in Biorender.com.

The *HLA* region (class I and II *HLA* genes) encodes the major histocompatibility complex (MHC), which is crucial for antigen presentation. Genetic variants in this region are commonly implicated in altered immune system recognition of autoantigens and susceptibility to autoimmune diseases [47,48]. From 29 genetic association studies, 32 *HLA* haplotypes have been identified to confer autoimmune disease risk (Figure 2), with several being shared across global populations. Several studies consistently highlight the significance of *HLA-DRB1* alleles in AIH susceptibility. Notably, haplotypes HLA-DRB1*04:01, *13:01, and *04:05 are the most common risk loci which have been identified in studies conducted in Europe, North America, and Asia.

The first AIH-associated genome-wide association study (GWAS) conducted on Dutch AIH patients showed the association of the AIH phenotype with the *HLA* region, particularly HLA-DRB1*03:01 and HLA-DRB1*04:01 genotypes (Table 1, Appendix A, Table A1) [49]. The HLA-DRB1*03 allele, associated with an earlier age of onset and higher IgG levels at presentation [49], further confers AIH disease risk in other European-ancestry cohorts [50,51] as well as West Indian [52], Thai [53], Venezuelan [54], Iranian [55], Tunisian [56], and North Indian populations [57]. The HLA-DRB1*04:01 allele, associated with later disease onset [49], has been linked to AIH susceptibility in European [58], North American [50,51], Chinese [59], and Japanese [60] cohorts. In addition, the HLA-DRB1*04:04 and DRB1*04:05 alleles are shown to be associated with AIH disease risk in Mexican [61], Argentinian [62], Japanese [60,63,64,65], Korean [66], and Latin American cohorts [67]. However, contradictory to North American and European studies, HLA-DRB1*04 was not significantly associated with AIH in an Italian patient population [68]. Collectively, this research underscores the central role and regional differences in *HLA* alleles in AIH susceptibility and clinical presentation.

*HLA* class I alleles, such as HLA-A*03:01, have been identified as protective alleles associated with reduced AIH susceptibility [50]. Protective alleles, such as HLA-DRB1*11, have also been found to be region-specific in a Southern European population [68]. Additionally, Oka et al. [60] and Duarte-Rey et al. [67] showed the protective association of DRB1*13:02 for AIH in Japanese and Latin American cohorts, respectively [60,67].

Prior research has defined *HLA* allele prevalence using allele frequency (AF) thresholds of >10% and >20%, revealing trends between global population groups and AIH risk loci [48,69]. The HLA-DRB1*04:01 allele (associated with AIH risk) is frequent (AF > 10%) in European, North East Asian, and North American regions [48,69]. This allele is also frequent in the North African, South West Asian, and South American regions but is associated to a lesser degree with AIH disease risk in these population groups [48,69]. Additionally, the HLA-DRB1*03:01 risk allele is frequent (AF > 10%) in global populations but was found to have a higher allele frequency (AF > 20%) in the North African region, supporting the AIH association found by Chaouali et al. [56] in Tunisia [48,56,69]. The HLA-DRB1*04:04 risk allele is not frequent (AF < 10%) in any of the global populations, whereas the DRB1*04:05 risk allele is very frequent (AF > 20%) in the Oceania region (including the Pacific, New Guinea, and Australia), where there is limited research into AIH genetic associations [48,69].

Elucidating the role of *HLA* alleles in disease risk has been hindered due to technical limitations, including the poor resolution of microarrays, high *HLA*-typing sequencing costs, and high linkage disequilibrium at *HLA* loci for the general population [48,50,69]. Additionally, there is a paucity of representative research on *HLA* genotypes (not restricted to association with disease) in many global populations as represented by the Immuno Polymorphism Database (IPD) IMGT/HLA Database—with under 0.04% of submissions from the African continent (all 15 submissions from South Africa), 1% from India, and 3.2% from South America, in comparison to the majority of submissions, over 80%, from North America (34,879 submissions) [47]. However, despite this lack of research, variations in the distribution of *HLA* alleles among different ethnicities have been noted, suggesting that a population-specific risk for autoimmune disease, including AIH, could be inferred, and highlighting the complexity of the genetic underpinnings of the disease.

**Table 1:** Genome-wide association studies (GWAS) conducted for autoimmune hepatitis.

Candidate Gene Variant Risk Allele	Gene Function	SNP Risk Allele Frequency ^1^ Global|Study Population|Africa	GWAS *p*-Value ^2^	Other Disease Associations	Ref.
*CARD10* rs6000782-C	Immune response and apoptosis	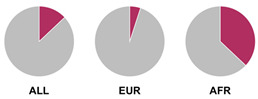	3 × 10^−6^	Prostate cancer Oesophageal varix Keloid	[49]
*SH2B3* rs3184504-A	Adaptor protein in immunity and proliferation signalling pathways	Rare variant is not present in major population-level allele frequency tables.	8 × 10^−8^	Hypothyroidism Type 1 diabetes Celiac disease	[49]
*HLA-DQA1* rs2187668	Antigen presentation	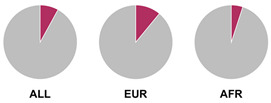	2 × 10^−78^	Ulcerative colitis Inflammatory bowel disease Rheumatoid arthritis	[49]
*CD28/CTLA4/ICOS* rs72929257-T	T cell activation and proliferation	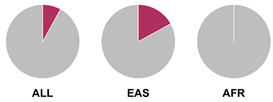	3 × 10^−9^	Graves’ disease Hearing loss Autoimmune disease	[70]
*SYNPR* rs6809477-A	Synaptic vesicle trafficking and neurotransmitter release	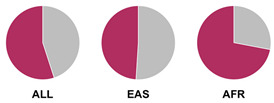	5 × 10^−9^	Rheumatoid arthritis Type 2 diabetes Intracerebral haemorrhage	[70]
*HLA-B* rs6932730-G	Immune response	Rare variant is not present in major population-level allele frequency tables.	9 × 10^−73^	Rheumatoid arthritis Autoimmune multi-trait Graves’ disease	[70]
*STAT1/STAT4* rs11889341-A	Cytokine signalling	Rare variant is not present in major population-level allele frequency tables.	1 × 10^−7^ (suggestive)	Rheumatoid arthritis Autoimmune multi-trait Autoimmune disease	[70]
*LINC00392* rs9564997-C	Gene regulation	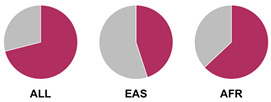	3 × 10^−7^ (suggestive)	Pancreatic cancer Uterine fibroid Myocarditis	[70]
*IRF8* rs11117432-A	Transcription factor for development of monocyte lineage cells	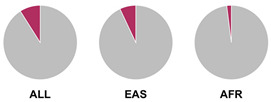	6 × 10^−6^ (suggestive)	Rheumatoid arthritis Allergy multi-trait Type 2 diabetes	[70]
*LILRA4/5* rs11084330-G	Regulation of myeloid cells	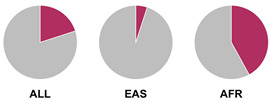	5 × 10^−6^ (suggestive)	Intracranial germ cell tumours Hunner-type interstitial cysts Allergy multi-trait	[70]
*TSBP1* rs56036302-T	Inflammation and immune responses	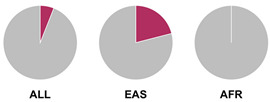	7 × 10^−9^	Rheumatoid arthritis Autoimmune disease Graves’ disease	[71]
*HLA-DQB1* rs1794514-C	Antigen presentation	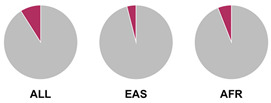	6 × 10^−14^	Autoimmune multi-trait Allergy multi-trait Rheumatoid arthritis	[71]

^1^ Allele frequency data from Ensembl the 1000 Genomes Project data, which is organised into global population (ALL) and subdivided into five superpopulations: Africans (AFR), Admixed Americans (AMR), East Asians (EAS), Europeans (EUR), and South Asians (SAS). Variant risk allele highlighted in maroon within pie charts. ^2^ GWAS data sourced from GWAS Catalogue from Trait Autoimmune Hepatitis (EFO_0005676). Data from all superpopulations represented in Appendix A, Table A1.

#### 3.1.2. Single-Gene Variants

While noting the importance of *HLA* alleles in autoimmune diseases, other single-gene variants have also been identified as risk alleles for AIH. The strongest GWAS signal identified in a Dutch study, rs3184504, mapped to the Src homology 2 adaptor protein 3 (*SH2B3)* gene, involved as a negative regulator of cytokine signalling and T cells [49]. The rs3184504-A (risk allele) is a missense variant with no reported clinical significance, with no allele frequency data from 1000 Genomes, and is only recorded in the TOPmed cohort at a 0.005 frequency. Similarly to the Dutch study, AIH disease association to the *SH2B3* rs3184504 polymorphism was validated in a candidate gene study from Tunisia, North Africa [72]. However, the disease-conferring association to *SH2B3* was not replicated by genotyping analysis in a Japanese cohort, which identified another *SH2B3* polymorphism, rs11065904, an expression quantitative trait loci (eQTL), hypothesised to be associated with AIH protection [73]. The GWAS conducted by de Boer et al. [49] additionally reported on the association of AIH with a Caspase Recruitment Domain Family Member 10 (*CARD10)* gene polymorphism, indicating that mechanisms related to cell death and signalling may contribute to liver inflammation and damage [49]. However, the association of AIH with the *CARD10* polymorphism was not validated in a single-gene study in a Japanese cohort [74].

The AIH risk-conferring genes identified by de Boer et al. [49] were not validated by a GWAS meta-analysis in a Han Chinese population, as illustrated in Table 1 [70]. However, these discordant results were attributed to population-level allele frequency differences. The strongest GWAS signal was a SNP within the *HLA* region (rs6932730), followed by significant signals from two novel SNPs (rs72929257 and rs6809477) (Table 1) [70]. Additionally, there was suggestive evidence for the association of the signal transducer and activator of transcription 4 (*STAT4*) with AIH [70], which was furthermore replicated in candidate gene studies for AIH and other autoimmune diseases [72,75].

Interestingly, the GWAS conducted by Li et al. [70] showed suggestive signals for long non-coding RNAs (lncRNAs) such as *LINC00392*, which has a proposed but unconfirmed role in gene regulation [70]. Additionally, transcriptomic analyses identified *LINC01089* (LncRNA Inhibiting Metastasis) to be upregulated in AIH, where gene enrichment analysis associated the lncRNA with the enrichment of the Mitogen-Activated Protein Kinase (MAPK) and Wnt signalling pathways [76]. Variants in this non-coding region suggest that regulatory elements outside of protein-coding genes may also be important in AIH susceptibility [70,76].

Notably, in candidate gene studies, a SNP in the tumour necrosis factor-alpha (*TNF-α*) gene (rs1800629) has been associated with increased AIH risk in diverse populations, including cohorts from New Zealand and Tunisia [77,78,79]. In contrast, studies in Brazilian and Mexican populations, where AIH is not strongly associated with *HLA-DRB1,* have shown no association of *TNF-α* polymorphisms with AIH [80,81].

Collectively, these findings highlight how dysregulation in immune signalling pathways plays a critical role in the development of AIH. Additionally, genes related to immune response modulation, such as protein tyrosine–protein phosphatase nonreceptor type 22 (*PTPN22)* polymorphisms may increase susceptibility or influence the clinical course of AIH [72]. However, some studies report a lack of association between AIH and other polymorphisms, such as transcription factor T-box expressed in T cells (*TBX21*) and Fc receptor-like gene 3 (*FCRL3*), indicating the complex and multifactorial nature of the disease [82,83].

### 3.2. Environmental Triggers

Environmental exposures can play a significant role in the development and progression of AIH, contributing to genetic predispositions to influence disease onset and severity. To date several key exposures have been hypothesised to precipitate autoimmune responses in the liver including infections, medications, and toxins [84,85].

The triggering of AIH by viral infections has been proposed, implicating molecular mimicry, the targeting of some autoantigens by the immune system due to their structural similarity to the pathogen, as the mechanism of autoimmunity. Pathogens including hepatitis C [86], hepatitis A [87], hepatitis E [88,89], and the Epstein–Barr virus [90] have been profiled in case reports; however, the incidence, molecular mechanism, and the potential role of genetic susceptibility for viral-triggered AIH is not completely understood [91]. Despite its recently established association with autoimmune diseases such as systemic lupus erythematosus [92], Epstein–Barr virus appears to have a protective role in AIH through the suppression of inflammatory pathways and immunomodulation of inflammatory cytokines [93]. Despite its broad effects on immune activation, Human Immunodeficiency Virus (HIV) has only infrequently been linked to AIH in the literature, and the underlying mechanisms remain uncertain [38,94,95]. Noting small patient sample sizes in AIH case studies (average *n*~3), most people living with HIV had undetectable viral loads, presented with elevated liver enzymes, and were able to achieve AIH remission through treatment [95,96]. In a cross-sectional database study in North America, just over 50 per 100,000 people living with HIV were diagnosed with AIH; however, in keeping with other AIH studies, the authors observed a predominance of females and African American patients [95].

Drug-induced AIH occurs when drug exposure triggers an immune-mediated response leading to liver damage, which has been documented in patients using, among other medications, antimicrobials such as nitrofurantoin and minocycline [2]. Occupational exposures and pollutants, such as pesticides and heavy metals, may also contribute to liver injury and subsequent autoimmune responses [2,97]. Moreover, lifestyle factors, including diet and alcohol consumption, can modulate immune function and influence the risk of AIH [85]. Critically, the gut–liver axis is an important component of immune signalling during liver disease as reviewed by Hsu & Schnabl [98]. This bidirectional axis of communication and circulation between the liver and gut microbiome can be altered by medication, diet, infection, and other environmental exposures [98]. Gut dysbiosis and increased intestinal permeability have been shown to influence AIH with earlier onset and increased disease severity [99,100,101,102,103,104,105]. Understanding these environmental influences is essential for identifying at-risk individuals, preventing disease onset, and developing strategies to mitigate the impact of these triggers.

### 3.3. Dysregulated Immune System

Autoimmune hepatitis pathogenesis has been associated with the dysregulation of the innate and adaptive immune cell populations. There are several cell types and mechanisms implicated in the hepatic destruction and loss of immunological tolerance which contribute to the disease.

#### 3.3.1. Innate Immune System

**Figure 3:**
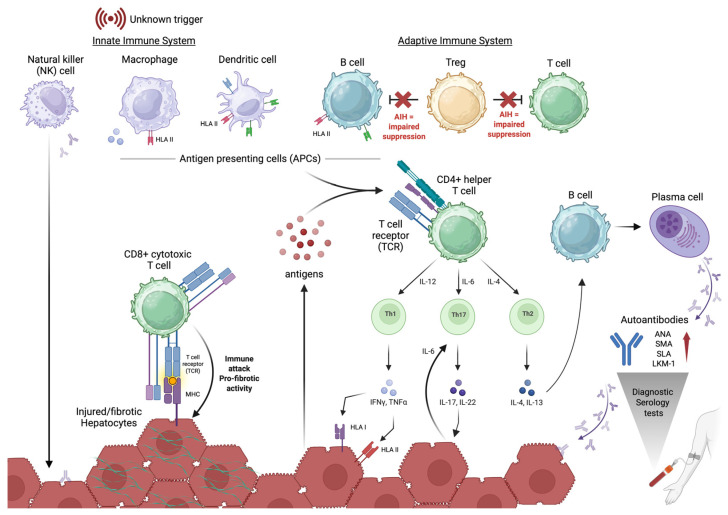
Mechanisms of hepatic destruction via dysregulation of the immune system during autoimmune hepatitis (AIH) pathogenesis, highlighting roles of the innate immune system, regulatory T cells (Treg), activated CD4+ T cells, cytotoxic CD8+ T cells, and B cells. The innate immune response is primarily activated by a trigger, leading to presentation of autoantigens to T cells. This results in aberrant activation of autoreactive CD4+ T cells, which in a feedback loop, release cytokines and B cells mature into autoantibody-producing plasma cells. Tregs, normally responsible for suppressing immune responses, are dysfunctional, allowing unchecked proliferation and activation of T and B cells. In combination, these factors drive chronic liver inflammation and hepatocyte injury. Figure created in Biorender.com.

The innate immune system comprises cell types like natural killer (NK) cells, macrophages, and dendritic cells, which can critically influence cellular homeostasis and consequently the adaptive immune response during AIH disease progression (Figure 3) [76,106,107,108,109,110].

NK cells regulate adaptive immunity through the enhancement and inhibition of T and B cell responses [111,112]. During AIH pathogenesis, cells presenting upregulated stress-induced ligands or MHC class I deficiency result in NK cell autoreactivity [107,109]. The released cytokines target hepatocytes, dendritic cells, and T cells exacerbating the disruption of immunological tolerance during AIH. Killer immune–globulin-like receptors (KIRs) and high-affinity *HLA* ligands have been shown to contribute to AIH-associated NK cell autoreactivity [109,111].

Macrophages and dendritic cells (DCs) form important immunological bridges from the innate to the adaptive immune system as antigen-presenting cells (APCs) and factories of co-stimulatory signals [113]. These cells can be activated in distinct spatial microenvironments in the liver, influencing cellular phenotypes and functionality—this heterogeneous context can complicate the ability to elucidate the role of specific cell subsets in diseases such as AIH [114,115].

During disease pathogenesis, the homeostatic ratio between M1 (pro-inflammatory) and M2 (anti-inflammatory) macrophages is disrupted. Although the mechanisms are not yet fully elucidated, in AIH this is associated with an infiltration of monocyte-derived M1 macrophages within the liver accompanied by the polarisation of liver-resident macrophages, Kupffer cells, to pro-inflammatory states [110,116,117]. The altered function of Kupffer cells in AIH, including impaired phagocytic ability impacting APC functionality, has been linked to differential expression of effectors/activators of the Rho family GTPases [108]. Furthermore, there is an aberrant interaction between Kupffer cells and hepatocytes, leading to the hepatic destruction seen in AIH histology [118].

Dendritic cells, in their sentinel capacity, act via antigen presentation and phagocytosis. Notably for AIH pathogenesis, DCs can uniquely activate naïve T cells. Although the effect on AIH pathogenesis is not fully understood, studies have demonstrated the alteration of DC cell type frequencies/proportions in AIH patients with the accumulation of peripheral mature DCs in AIH patients being associated with increased disease severity [76,106]. Conversely, the proportion of peripheral plasmacytoid DCs was shown to be lower in AIH patients in comparison to healthy controls—demonstrating the potentially protective role of this subset of DCs [119,120].

#### 3.3.2. T Cell-Mediated Immunity

The pathogenic mechanism of AIH is underpinned by T lymphocytes (T cells), particularly CD4+ helper T cells and CD8+ cytotoxic T cells [121,122].

Also known as helper T (Th) cells, CD4+ T cells and their subsets are upregulated during the pathogenesis of AIH [113,123,124,125,126,127]. CD4+ T cells are activated through T cell receptors (TCRs) that recognise liver-specific antigens presented by *HLA* II on APCs—a molecular mechanism that is proposed to correspond to the *HLA* allele disease-conferring risk for AIH [126,128,129]. Once activated, specific antigen recognition leads to CD4+ T cell differentiation into subsets, including Th1, Th2, Th17, and Th22, which have distinct cytokine profiles and activate other immune cell types [78,113,125,130]. During the pro-inflammatory pathogenesis of AIH, upregulated Th1 cells are characterised by the secretion of interferon–gamma (IFNγ), which stimulates cytotoxic T cells [121,122,124,131]. Th2 cells secrete interleukin (IL)-4, IL-13, and IL-21, promoting the activation of B cells into plasma cells and upregulating autoantibodies [113,125]. Th17 cells (derived in the presence of IL-6) produce IL-17 and TNF-α which in turn form a positive feedback loop with the expression of IL-6 by hepatocytes [123,132]. In AIH patients, the persistent activation of circulating T cells has been shown to contribute to ongoing liver inflammation and damage [126,128]. Furthermore, the CD4+ cytokine cascade and feedback loops perpetuate disease-mediated hepatocyte injury while impairing the function of regulatory mechanisms contributing to the pathways implicated in AIH pathogenesis.

Helper CD4+ T cells are implicated in the initial autoimmune response, however, autoantigens presented by MHC class I on hepatocytes are recognised by TCRs on cytotoxic CD8+ T cells which, once activated, induce hepatic injury and fibrosis and release pro-inflammatory cytokines (Figure 3), further enabling a persistent inflammatory state in the liver [121,122,131,133].

#### 3.3.3. B Cell-Mediated Immunity

The multifaceted B lymphocyte (B cells) play a role in AIH pathogenesis by maturing into plasma cells (in secondary lymphoid tissue) and are responsible for increased autoantibody production—a hallmark for AIH diagnosis [4,127,134,135]. The role of B cells in the presentation of autoantigens to undifferentiated T cells is considered a driver of autoimmunity, and B cell depletion was shown to modulate T cell proliferation and function [136]. However, a recent study by Lübbering et al. [137] showed that activated B cells can act in a “bystander” capacity, as T cell-mediated hepatitis recapitulated in a mouse model was independent of B cell presentation [137]. Additionally, immunophenotyping studies have shown no evidence of B cell-specific signatures in AIH patients (regardless of immunosuppression), emphasising their potential role in mediating instead of fuelling the AIH immune response [138,139]. While these studies elucidate the complementary role of these lymphocytes, B cell-targeting therapies (such as rituximab) have been effective in patients who are refractory to standard treatment, illustrating their probable role in perpetuating T cell activation [136,140].

#### 3.3.4. Regulatory T Cells

The absence or dysfunction of Tregs has been ubiquitously implicated across autoimmune diseases and is critical to AIH pathology through the loss of immune tolerance and inability to suppress autoreactive T and B cells [122,131,134,141,142]. The depletion of Tregs (notably, a consequence of immunosuppression therapy for AIH) is correlated with markers of the decompensated AIH phenotype [122,131,134,141]. Additionally, upregulating Tregs through treatment with IL-2 showed normalisation in liver enzymes in mice, illustrating the regulatory role in AIH pathogenesis [143]. Although the exact mechanism of Treg dysregulation is not fully understood for AIH, the combinatorial effect of the expansion of autoreactive cells and increased presence of autoantibodies contributes to the chronic inflammation observed [134,141,144,145].

### 3.4. Transcriptomics in Deconvolving Molecular Profiles of AIH

Technological advances in the molecular profiling of biological samples are enabling unprecedented insights into health and disease. The application of transcriptomic profiling (RNA sequencing) in AIH has begun to reveal the molecular basis of immune system dysregulation. Several studies have reported the upregulation of genes linked to immune activation, inflammation, and antigen presentation, emphasising the roles of T cells, B cells, and antigen-presenting cells in AIH pathogenesis as explored above [126,146,147]. Transcriptomics analysis of whole blood or isolated innate immune cells (monocytes and NK cells) revealed the upregulation of the interferon-mediated signalling pathway in both the innate (specifically linked to IFN-γ) and adaptive immune cells, indicative of a heightened immune response in AIH patients [146,147]. Furthermore, whole blood analysis revealed a downregulation of CD8+ cell-related gene expression in AIH patients [147].

The application of single-cell RNA sequencing (scRNA-seq) has provided a more detailed understanding of the cellular heterogeneity within AIH, identifying specific immune cell subsets, gene expression profiles, and cellular interactions that contribute to disease development. Abe et al. [146] generated scRNA-seq profiles of peripheral blood mononuclear cell (PBMC) samples of four female AIH patients and four healthy sex-matched controls [146]. Analysis of gene expression across seven thousand single cells revealed 16 cell type clusters with marker gene expression associated with immune-derived hematopoietic cells [146]. Notably, the enrichment of effector CD8+ T cells, monocytes, NK cells, and naïve CD4+ T cells was significantly altered in AIH compared to controls. Specifically, effector CD8+ T cells were enriched in AIH by 6% in comparison to healthy controls—consistent with the cytotoxic lymphocyte-driven hepatic damage during AIH pathogenesis (Figure 3) [146]. Conversely, a 5% depletion of naïve CD4+ T cells was observed in AIH samples versus healthy controls—indicative of chronic T cell activation and expansion during AIH progression [146]. However, all patients enrolled in the study were treated with a combination of immunosuppression therapies, which would negatively affect the proportion and type of inflammatory cells present in the blood [146]. Differential gene expression analysis revealed a higher number of differentially expressed genes (DEGs) in monocytes (87 up- and 12 downregulated) and NK cells (101 up- and 15 downregulated) to be associated with AIH in comparison to the other 14 cell types (<25 DEGs per cell type) [146]. Gene Ontology (GO) enrichment analysis uncovered an association with antigen presentation, IFN-γ signalling, and neutrophil activation [146]. A similar study, focused on AIH transcriptomic profiles of PBMCs, investigated soluble liver antigen-specific autoreactive CD4+ T cells [126]. Active AIH was associated with a distinctive phenotype of memory CD4+ and CD8+ T cells (CD45RA−PD-1 + CD38 + CXCR5−CD127−CD27+), which, through the production of IL-21, stimulates B cell differentiation [126]. Furthermore, an association between the T cell subset frequency and transaminase levels was identified—an indicator of liver parenchymal cell destruction and potentially clinically predictive of relapse during remission [126]. The expansion of Tregs and upregulation of interleukin-7 receptor (*IL-7R*) was demonstrated through scRNA-seq (*n* = 1) and further confirmed by flow cytometry (*n* = 45) in PBMCs from Korean AIH patients versus healthy controls [142]. Furthermore, functional assays illustrated that although the number of Tregs increased in response to hepatic inflammation, the suppressive functionality was impaired in both peripheral and hepatic samples, hypothesised to lead to B cell and T cell expansion (Figure 3) [142]. Future research, in larger patient cohorts, is proposed to characterise TCR repertoires and elucidate the role of Treg alterations in AIH pathogenesis [142].

The use of peripheral blood in these studies provides insights into the systemic regulation of AIH, but not the cellular interactions resulting in progressive tissue destruction in the liver [138,146,147]. Liver biopsies, although rare in research settings, are valuable for the investigation of hepatic gene expression and immune dysfunction associated with AIH. A recent AIH-focused study captured over 45 thousand single cells from four diseased liver biopsy tissue samples and two controls, from which the authors identified six major cell types [148]. Myeloid cluster subtype analysis revealed that the macrophage migration inhibitory factor (MIF) acts as a key upstream inflammatory factor in AIH [148]. This is hypothesised to result in the secretion of other pro-inflammatory cytokines such as IL-6, which activates CD4+ T cells, forming a positive feedback loop and promoting hepatic inflammation (Figure 3). Additionally, two immune cells, CD8-Tc-PDCD1 and CD8-Tc17-RORc, are highlighted as potential modulation targets of MIF [148].

The continual advancement of gene expression technology, including single-cell and spatial transcriptomics, shows the promise of uncovering previously unknown disease pathogenesis and mechanisms. Integrating additional layers of molecular information, such as epigenomics, proteomics, and metabolomics, could further refine the understanding of autoimmune liver disease pathogenesis and identify novel therapeutic targets.

### 3.5. Animal Models to Understand the Pathophysiology of AIH

The development of several AIH-focused animal models has been instrumental in advancing the understanding of the disease pathophysiology. Among the most researched is the standard Concanavalin A (ConA)-induced hepatitis mouse model, which replicates acute liver inflammation by targeting T cell activation. Studies utilising the ConA model have revealed the critical role of CD4+ and CD8+ T cells in mediating liver injury and highlighted the importance of cytokines like TNF-α and IFN-γ in driving the inflammatory response [149,150,151,152]. Additionally, the mechanisms of Treg dysfunction in AIH have been investigated, demonstrating how a loss of immune tolerance leads to the uncontrolled activation of autoreactive T cells. However, these insights are accompanied by the acknowledgement of the limitations of in vivo models, where the human hepatic environment, disease initiation, and knock-on immune dysregulation cannot be recapitulated [153,154]. Therefore, studies have leveraged techniques in both human patient samples and ConA AIH-induced mouse models to investigate the dysregulation of differentiated helper T cells (Th17) due to the inactivation of the aryl hydrocarbon receptor (AhR) [155,156].

Insights from mouse model research have supported the use of standard immunosuppression regimens, including corticosteroids and azathioprine. However, alternative therapeutics such as anti-folate drugs (e.g., pemetrexed) and pomegranate peel have also shown therapeutic potential [157,158]. Preclinical findings have explored specific immune pathways such as TNF-α blockades and anti-cytokine therapy with IFN-γ small interfering RNA (siRNA) [151,159,160]. Additionally, ConA-induced hepatitis was alleviated in mice through the administration of siRNA exosomes targeting receptor-interacting protein kinase 3—implicated in cell death signalling [161,162]. Liver-targeted treatments for fibrosis, cancer, and viral hepatitis using antisense oligonucleotides have been explored, with an example of the mechanism of action for fibrosis involving the degradation of the extracellular matrix and activation of hepatic stellate cells [163]. However, the use of these therapies has not yet been extended to autoimmune liver diseases [163].

Transcriptomic analysis of human PBMCs and liver biopsies has provided detailed insights into the disease landscape of AIH; however, murine models offer a controlled system to explore mechanistic drivers in vivo and to identify putative therapeutic targets. Single-cell transcriptomic research has further demonstrated the mitigation of ConA-induced AIH through AhR activation and subsequent cytokine regulation, which allowed for the identification of a potential therapeutic agent—tetrachlorodibenzo-p-dioxin [164]. Additional agents aimed at therapeutic intervention have been identified from scRNA-seq data using the CoA murine model, such as astaxanthin, which is proposed to modulate CD8+ T cells [165].

Integrating findings from animal models has been instrumental in advancing the understanding of the pathophysiology of AIH. These models mimic various aspects of human disease, allowing researchers to investigate underlying immune mechanisms, identify potential therapeutic targets, and test the efficacy of new treatments. However, the ConA mouse model requires artificial sensitisation and therefore lacks the ability to characterise the spontaneity and chronic onset of disease. The further utilisation of humanised Bone Marrow–Liver–Thymus mice models, which harbour a nearly complete human immune system, and gnotobiotic or faecal microbiota transplantation mice investigating the gut–liver axis, could provide valuable insights into autoimmune liver disease [166,167,168,169]. While acknowledging the drawbacks of studying human disease through model organisms by replicating key features of AIH, such as liver inflammation, autoantibody production, and immune cell infiltration, animal models provide a useful platform for investigating discrete research questions; however, given the differences between murine and human immune systems and physiology, the utility of these models for addressing complex interactions may be limited. Therefore, the use of immune-competent in vitro platforms, including cell lines, hepatic spheroids/organoids, and microfluidic systems, could support translational research in the multifactorial and complex disease pathogenesis of AIH [153,154].

## 4. Global Population Research for Autoimmune Hepatitis

Research on AIH has historically been predominantly represented by populations of European ancestry in developed countries, with research in Africa, Asia and South America being limited by small cohort sizes and a lack of suitable control groups. Extensive research is needed to understand the intricacy of AIH in underrepresented population groups to improve diagnosis, treatment, and long-term outcomes.

Recent findings illustrated that African American patients in North America were significantly more likely to be diagnosed with AIH in comparison to patients of European ancestry who were more likely to be diagnosed with MASLD [170]. Several additional studies from North American institutions have also shown that African American and Hispanic patients with AIH often present with more severe liver inflammation and fibrosis at diagnosis compared to patients of European ancestry [14,40,171,172,173,174]. Additionally, a nationwide study found that hospitalisation of African American patients for AIH was associated with a significantly higher rate of mortality [11]. The more aggressive disease course may be due to differences in genetic risk linked to single-gene variants as well as *HLA* profiles, the latter of which can consequently result in variations in immune system dysfunction across ethnicities. Furthermore, delayed diagnosis due to disparate access to healthcare and socioeconomic status may impact epidemiology statistics and mortality rates.

Geographical and socioeconomic disparities in healthcare access and quality also significantly impact AIH outcomes due to the requirement for diagnostic biopsy and expensive long-term immunosuppressive schedules [4,14,175]. In under-resourced regions or among historically underserved populations, there may be delays in diagnosis due to limited access to specialised healthcare, leading to more advanced disease presentation [172]. Socioeconomic factors, such as lower income and educational levels, are often confounded by ethnic associations and can further exacerbate these disparities, contributing to poorer long-term outcomes for patients from certain ethnic backgrounds.

### A Continental African Perspective

There is a paucity of epidemiological data on liver diseases, such as AIH, from Africa with the status of healthcare infrastructures often limiting the centralisation of electronic information required for registries—with specialised liver transplant clinics in only two countries (South Africa and Egypt) on the continent. The lack of resources dedicated to liver disease research has been further limited by the focus on the urgent and overwhelming burden of infectious diseases in comparison to lower-prevalence autoimmune conditions [12,17].

Diagnostic criteria for AIH has been refined into a simplified scoring system for use in clinical practice settings [1,3,176]. Investigations into the diagnostic value of the simplified criteria have highlighted the critical nature of including liver histology in combination with clinical and serological results [177,178,179,180]. However, there are few validation studies investigating the sensitivity and specificity in global population groups, with no published data available from African patient populations [177,178,179,180]. In a South African transplant setting, 33% of transplant recipients with an indication for AIH did not have AIH on explant histology, indicating a need to increase the sensitivity of the diagnosis in the local population [181]. However, the International AIH Group simplified scoring system continues to be utilised by local clinicians despite the limited validation. In another South African cohort, the stratification of the majority (62.5%) of patients into probable AIH and only 37.5% as definite AIH demonstrates a need to increase the specificity between the diagnostic sub-categories [38].

Considering these factors, anecdotal evidence and emerging studies suggest that AIH may be more common in Africa than reported, and research has therefore focused on characterising the clinical presentation and outcomes of AIH in specific African regions [38,181,182,183]. However, these studies are limited in sample size, ranging from single-case reports (*n* = 1) from Nigeria [184,185] to small cohort studies in South Africa (*n* = 40) and Malawi (*n* = 5) [38,186]. The most recent retrospective study of AIH in a South African hospital displayed the expected female predominance in the cohort; however, in contrast to Northern global populations, an earlier age of onset (26 years) was calculated [38]. Additionally, the AIH cohort had a higher prevalence of cirrhosis (50%), illustrating the potential early and aggressive disease onset pattern [38]. The autoantibody profile reported for the general cohort was compatible with diagnostic guidelines; however, the elevated cholestatic enzymes and low albumin (34 g/L) may suggest an advanced or overlapping disease presentation [38]. Notably, the treatment response and transplant statistics were not reported in this study. Although the cohort predominantly comprised Black African patients, the small sample size limited comparisons of disease progression between population groups [38]. However, analysis of the diagnostic and presenting features revealed that Black African patients often present with more advanced disease compared to their European counterparts—possibly due to delayed diagnosis.

Although there have been reports of AIH potentially triggered by viral agents, these are limited and the etiological association has not been fully explored. In Africa, the high burden of infectious diseases, such as HIV, tuberculosis, and hepatitis B, further complicates the clinical landscape of AIH [12,187]. These infections can mimic or exacerbate autoimmune liver disease, leading to diagnostic challenges and delayed treatment [38]. In particular, the co-occurrence of HIV and AIH presents a significant challenge, as the immunosuppressive treatment required for AIH may have adverse implications for individuals with compromised immune systems [188,189]. Maharaj & Naidoo [38], observed 40% of the AIH cohort from South Africa was comprised of people living with HIV (all on highly active antiretroviral therapy with suppressed viral loads). Comparing AIH patients living with and without HIV [38], in a cohort of 33, revealed a trend of higher prevalence of cirrhosis and portal hypertension as well as lower transaminase levels in AIH patients living with HIV [38]. The intersection of infectious and autoimmune diseases necessitates careful consideration in clinical practice, with a need for tailored treatment protocols that account for the complexities of managing AIH in the context of concurrent infections.

The outcomes of liver transplantation provide an insight into disease severity as well as the recurrence of disease, which is specifically important for autoimmune liver diseases. A study by Siddiqui et al. [181] focused on the outcomes of liver transplantation for AIH in South Africa, highlighting both the successes and challenges in managing end-stage AIH in a resource-limited setting. The authors observed that 78% of the cohort were female, 42% were Black African, and the average age of transplant was 37 years. In addition, high-risk populations for rejection were identified, with 70% of rejections occurring in Black African patients [181]. Although overall survival rates in this South African cohort were comparable to international cohorts, the researchers observed a 30–lower five-year graft survival rate and a higher rate of recurrence of AIH post-transplant in Black African individuals compared to their European-ancestry counterparts [181].

Noting the influential differences in environment and genetics in North African countries such as Tunisia and Egypt, there is limited data available on the epidemiology of AIH in this region. However, AIH was reported in a cohort of patients with acute hepatitis (*n* = 103) in Tunisia [190]. In a small cohort (*n* = 18) of Egyptian adults, AIH was shown to be the third most common cause of acute hepatitis (11% of patients with known hepatitis diagnosis), with similar statistics to hepatitis E virus (11%) and drug-induced liver injury (22%) [191].

Considering the regional disparities observed in the above African-based studies on AIH, there may be several factors that may influence the geographical distribution of AIH in Africa. Genetic predispositions, including specific *HLA* haplotypes and single-gene variants, are hypothesised to play a role in regional differences in disease prevalence [192]. Notably, genomic research needs to be cognisant of Africa’s vast genetic diversity, in comparison to that of European-ancestry populations, when investigating the genetic aetiology of a complex disease such as AIH [193,194,195]. Additionally, the difference in genetic variation between African countries and within local ethnolinguistic groups [196,197], complicates the extrapolation of genetic findings in discrete population groups to the entire African continent.

Contrary to previous studies showing increased *HLA* allelic diversity in African cohorts, recent analysis illustrated low diversity within the Black South African population, comparable to the local European-ancestry population [192,198]. Further inter- and intra-country comparisons in Eastern and Southern Africa illustrated that the pattern of *HLA* allele frequency was not solely correlated to shared geographical location at the country and tribal level [199]. South Africa was the most divergent from other countries while Kenya had the greatest variation within the country’s borders [199]. When investigating single-gene variation, there are ongoing efforts to increase large-scale GWAS and multi-omic studies in African populations; however, there is little data available focused on autoimmune or liver diseases. A multivariate GWAS performed in Uganda (Eastern Africa) and South Africa (Southern Africa, Zulu cohort) demonstrated shared genetic loci associated with liver biomarker levels despite the considerable geographical distance between project sites [200]. Multi-cohort GWAS research has further identified novel significant variants linked to liver biomarkers—of which several had high allele frequencies in African populations but were rare in other study populations [201]. Considering these regional differences, disease-specific genetic association studies are required to fully elucidate the influence on AIH susceptibility and phenotype in different African countries.

Additionally, environmental factors, such as exposure to pathogens, gut microbiome dysbiosis, as well as medical prescription use, might also contribute towards AIH disease risk on the African continent [12]. In comparison to North America and Europe, there is a higher prevalence of endemic infectious and chronic diseases (e.g., HIV, tuberculosis, and malaria) in African regions, which shapes immune profiles and immunological profiles and memory [38,95,96,188,189,202]. In addition, more frequent exposure to antimicrobial medication, herbal supplements, or traditional remedies, can lead to drug-induced AIH [1,2,203,204]. The use of prescription medication, dietary habits and other built-environment factors can further influence the gut microbiome, with African cohorts demonstrating diverse microbial profiles, potentially impacting disease [205]. In a North African AIH cohort (*n* = 15), significantly lower bacterial diversity was observed compared to controls (*n* = 10), although the associated affected pathways were not related specifically to AIH pathogenesis or disease state [100].

Collectively, the AIH-focused studies that have been conducted in Africa highlight the need for increased awareness, early detection, and the development of region-specific management strategies to improve outcomes for AIH patients. These critical clinical strategies require an understanding of the pathogenesis of disease across local population groups, where currently there is a paucity of research with little-to-no research published from the African continent. Some studies have investigated the pathogenesis of autoimmune hepatitis in African American populations [11,14,171,172,173]. However, there may be differences in disease progression and environmental triggers between continental and African-ancestry populations, highlighting the limitations in extrapolating research findings from North America or Europe to represent population groups across the African continent.

## 5. Conclusions

The molecular mechanisms of AIH involve a complex interaction of environmental triggers, genetic predisposition, and immune dysregulation, leading to chronic inflammatory responses within the liver. Significant progress has been made in understanding AIH pathogenesis through genetic, cellular, transcriptomic, and model organism research. However, further research—particularly in the African and Asian context—is essential to fully elucidate the intricate mechanisms driving AIH across diverse global population groups. Collaboration within multi-disciplinary teams, including clinical, molecular biology, and public health specialists, is required to enable the integration of clinical multi-omic research with comprehensive electronic health records. This will support enhancements in diagnostic and treatment strategies which are relevant to historically underserved population groups and improve the clinical outcomes for AIH patients globally.

## Data Availability

The data presented in this study were derived from the following resources available in the public domain: GWAS Catalog at https://www.ebi.ac.uk/gwas/home, (accessed on 25 March 2025) Ensembl 1000Genomes at https://www.ensembl.org/ (accessed on 25 March 2025) and https://www.internationalgenome.org (accessed on 28 April 2025), and Immuno Polymorphism Database (IPD) IMGT/HLA Database at https://www.ebi.ac.uk/ipd/imgt/hla/ (accessed on 20 September 2025).

## Abbreviations and Gene Symbols

The following abbreviations are used in this manuscript:

AF	allele frequency
AhR	aryl hydrocarbon receptor
AIH	autoimmune hepatitis
*ANGPT2*	angiopoietin-2
APC	antigen presenting cell
DC	dendritic cell
DEG	differentially expressed gene
B cell	B lymphocyte
*CARD10*	caspase recruitment domain family member 10
*CD28*	cluster of differentiation 28
*CTLA4*	cytotoxic T-lymphocyte associated protein 4
ConA	concanavalin a
eQTL	expression quantitative trait loci
*ERα*	oestrogen receptor alpha
*FOXP3*	forkhead box P3
*FCRL3*	Fc receptor-like gene 3
GWAS	genome-wide association study
*HGF*	hepatocyte growth factor
HIV	human immunodeficiency virus
*HLA*	human leukocyte antigen
*HLA-B*	major histocompatibility complex, class I, b
*HLA-DQA1*	human leukocyte antigen-DQ Alpha 1
*HLA-DQB1*	major histocompatibility complex, class II, DQ Beta 1
*ICOS*	inducible T-cell costimulator gene region
IFN-γ	interferon-gamma
IL-7R	interleukin-7 receptor
IL-17	interleukin-17
*IRF8*	interferon regulatory factor 8
*LILRA4*	leukocyte immunoglobulin-like receptor a4
*LILRA5*	leukocyte immunoglobulin-like receptor a5
*LINC00392*	long intergenic non-protein coding RNA 392
lncRNAs	long non-coding RNAs
MAPK	mitogen-activated protein kinase
MASLD	metabolic dysfunction–associated steatotic liver disease
MHC	major histocompatibility complex
NK	natural killer cells
PBMC	peripheral blood mononuclear cell
*PTPN22*	protein tyrosine–protein phosphatase nonreceptor type 22
RNA	ribonucleic acid
scRNA-seq	single-cell RNA sequencing
*SH2B3*	sh2b adaptor protein 3
SNP	single nucleotide polymorphism
*STAT1*	signal transducer and activator of transcription 1
*STAT4*	signal transducer and activator of transcription 4
*SYNPR*	synaptoporin
*TBX21*	transcription factor T-box expressed in T cells
T cell	T lymphocyte
TCR	T cell receptor
Th	helper T cells
*TNF-α*	tumour necrosis factor-alpha
Tregs	regulatory T cells
*TSBP1*	testis expressed basic protein 1

## Appendix A

**Table A1:** Autoimmune hepatitis risk loci identified from GWAS with risk allele frequency in 1000Genome global population groups (Ensembl).

Candidate Gene Region	Variant, Risk Allele and Consequence	SNP Risk Allele Frequency in Global Population Groups ^1^	Ref
Caspase Recruitment Domain Family Member 10 (*CARD10*)	rs6000782-C intergenic variant		[49]
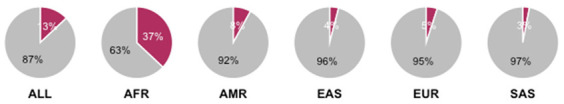
SH2B Adaptor Protein 3 (*SH2B3*)	rs3184504-A missense variant	Rare variant is not present in major population-level allele frequency tables.	[49]
Human leukocyte antigen-DQA1 (*HLA-DQA1*) Imputation: DRB1*0301/0401 genotype	rs2187668 intronic variant		[49]
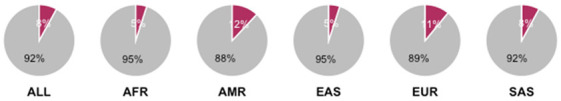
Cluster of differentiation 28/CTLA4/inducible T cell costimulator gene region (*CD28/CTLA4/ICOS*)	rs72929257-T intergenic variant		[70]
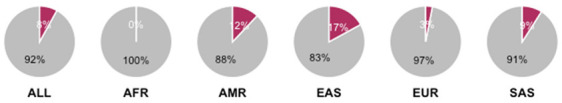
Synaptoporin (*SYNPR*)	rs6809477-A intronic variant		[70]
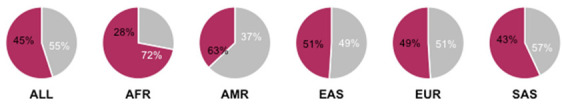
Major Histocompatibility Complex, Class I, B (*HLA-B*)	rs6932730-G intergenic variant	Rare variant is not present in major population-level allele frequency tables.	[70]
Signal Transducer and Activator of Transcription 1/4 (*STAT1/STAT4*)	rs11889341-A intronic variant	Rare variant is not present in major population-level allele frequency tables.	[70]
Long Intergenic Non-Protein Coding RNA 392 (*LINC00392*)	rs9564997-C intergenic variant		[70]
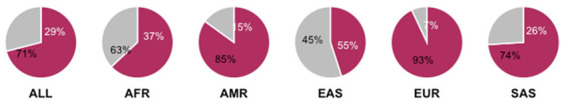
Interferon regulatory factor 8 (*IRF8*)	rs11117432-A intergenic variant		[70]
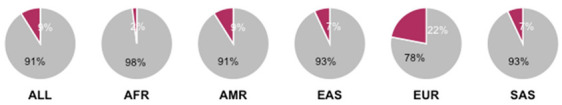
Leukocyte Immunoglobulin-Like Receptor A4/5 (*LILRA4/5*)	rs11084330-G 3′ UTR variant		[70]
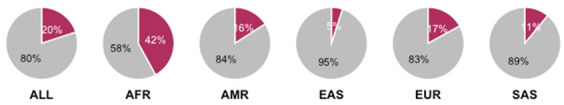
Testis expressed basic protein 1 (*TSBP1*)	rs56036302-T intronic variant		[71]
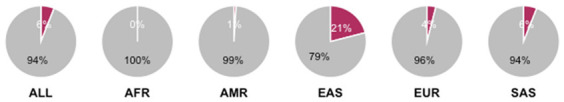
Major Histocompatibility Complex, Class II, DQ Beta 1 (*HLA-DQB1*)	rs1794514-C intergenic variant		[71]
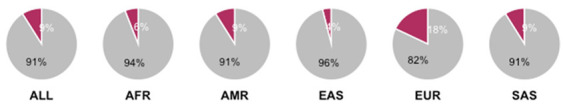

^1^ Allele frequency data from Ensembl the 1000 Genomes Project data, which is organised into global population (ALL) and subdivided into five superpopulations: Africans (AFR), Admixed Americans (AMR), East Asians (EAS), Europeans (EUR), and South Asians (SAS). Variant risk allele highlighted in maroon within pie charts.
